# Skin, thermal and umbilical cord care practices for neonates in southern, rural Zambia: a qualitative study

**DOI:** 10.1186/s12884-015-0584-2

**Published:** 2015-07-16

**Authors:** Emma Sacks, William J. Moss, Peter J. Winch, Philip Thuma, Janneke H. van Dijk, Luke C. Mullany

**Affiliations:** Department of International Health, Johns Hopkins School of Public Health, 615 North Wolfe Street, E8011, Baltimore, MD 21205 USA; Department of Epidemiology, Johns Hopkins Bloomberg School of Public Health, 615 North Wolfe Street, E6547, Baltimore, MD 21205 USA; Department of International Health, Johns Hopkins Bloomberg School of Public Health, 615 North Wolfe Street, E5533, Baltimore, MD 21205 USA; Macha Research Trust, PO Box 630 166, Choma, Zambia; USAID Maternal and Child Survival Program (MCSP)/ICF International, Washington, DC USA

**Keywords:** Newborn health, Neonatal health, Traditional practices, Skin care, Thermal care, Cord care, Zambia

## Abstract

**Background:**

In Choma District, southern Zambia, the neonatal mortality rate is approximately 40 per 1000 live births and, although the rate is decreasing, many deliveries take place outside of formal facilities. Understanding local practices during the postnatal period is essential for optimizing newborn care programs.

**Methods:**

We conducted 36 in-depth interviews, five focus groups and eight observational sessions with recently-delivered women, traditional birth attendants, and clinic and hospital staff from three sites, focusing on skin, thermal and cord care practices for newborns in the home.

**Results:**

Newborns were generally kept warm by application of hats and layers of clothing. While thermal protection is provided for preterm and small newborns, the practice of nighttime bathing with cold water was common. The vernix was considered important for the preterm newborn but dangerous for HIV-exposed infants. Mothers applied various substances to the skin and umbilical cord, with special practices for preterm infants. Applied substances included petroleum jelly, commercial baby lotion, cooking oil and breastmilk. The most common substances applied to the umbilical cord were powders made of roots, burnt gourds or ash. To ward off malevolent spirits, similar powders were reportedly placed directly into dermal incisions, especially in ill children.

**Conclusions:**

Thermal care for newborns is commonly practiced but co-exists with harmful practices. Locally appropriate behavior change interventions should aim to promote chlorhexidine in place of commonly-reported application of harmful substances to the skin and umbilical cord, reduce bathing of newborns at night, and address the immediate bathing of HIV-infected newborns.

## Background

To meet global targets for child and infant mortality reduction, the number of infants who die within the first 28 days of life must be reduced and the rate of progress accelerated [1, 2]. Over 40 % of child mortality occurs during the first month of life with almost one-third of these deaths attributed to infections [1, 3]. Another third of these 3.1 million deaths worldwide are caused by complications of preterm birth and low birth weight. Low birth weight infants are especially vulnerable to infection and hypothermia and require additional care during the first weeks of life [4]. HIV-exposed newborns are at increased risk of morbidity and mortality, and are more likely to be born low birth weight and preterm. [5, 6]. It is estimated that more than half of births in rural Zambia occur in the home, although recent government commitment to improve obstetric care facilities and train skilled attendants is expected to reduce the percentage of home births in the near future in at least some districts [7, 8]. Appropriate home-based implementation of care practices that reduce the risk of infection and hypothermia can contribute to significant reductions in neonatal mortality for infants born at home or in facilities if introduced and negotiated in an acceptable way [2]. Recommended practices include drying, head covering, delayed bathing and cord clamping, and cord cleansing with antiseptics; extra thermal care and topical emollient therapy is now recommended for low birth weight and preterm infants [9]. It is estimated that universal coverage and uptake of these key interventions would avert over half of current neonatal deaths [2, 9].

Studies in a number of countries have shown the effectiveness of providing care for newborns in the home shortly after birth [10, 11]. In Ghana and Tanzania, recent cluster-randomized trials have demonstrated effectiveness in significantly increasing uptake of key essential newborn care behaviors through home visits by community health workers [12, 13]. A meta-analysis calculated a potential reduction in the neonatal mortality rate of 12 % through home-based care packages [14]. Interventions effective at reducing the risks of neonatal infection and illness include early and exclusive breastfeeding, hand-washing by care provider and mother, thermoregulation of the newborn, neonatal vitamin A supplementation and clean cord care with chlorhexidine [15, 16]. Some single interventions can reduce multiple risks: delayed bathing may reduce the risk of infection and hypothermia, and skin-to-skin care may be beneficial for thermoregulation, breastfeeding, and reduction of infection and primary apnea [17, 18]. Many of these interventions are even more effective in protecting the health of preterm and low birth weight infants, who have underdeveloped lungs, skin barrier function, and immune defenses [18].

Targeting behavior change at the household level has repeatedly been identified as a potential strategy for improving newborn care [19, 20] and previous models have identified points of entry where programs can influence behavior change at the household or community levels [21, 22]. Much neonatal health research on home-based care practices by parents and traditional caregivers has been conducted in South Asia [15, 19, 23], with several recent studies from sub-Saharan Africa [24–28], including studies specifically focused on thermal care [29–31] and umbilical cord care [28]. However, the studies in Africa did not examine general skin and thermal care practices in the context of HIV endemicity, identify differing practices between infants thought to be HIV-exposed or not, nor differentiate between care of term and low birth infants. This study presents findings on neonatal skin, thermal and umbilical cord practices in an HIV-endemic region of rural southern Zambia.

## Methods

Choma District, in Southern Province, Zambia is 380 km from the capital city Lusaka. The population is approximately 135,000 within a 35 km radius around central Macha, a large chiefdom with a hospital and market [32]. Thirteen rural health centers within the catchment area refer patients to the hospital. The closest town, Choma, is 80 km away and has a general hospital, private clinics and pharmacies. In 2012, it was estimated that the neonatal mortality rate in Zambia was 34 per 1000 live births [33], and the HIV prevalence among adults was approximately 13 % [33].

The study was conducted between 2010 and 2011 and included interviews, focus groups and home observations. Participants were sampled from three areas: villages in the vicinity of Macha Hospital, a set of villages 5 km from the hospital and a set of villages greater than 15 km from the hospital. Traditional birth attendants were known to and identified by key informants, and were defined as individuals who had assisted in a home birth in the previous year, but had not received formal medical training. Recently-delivered women were identified by traditional birth attendants, community health workers and key informants. Participants were purposively sampled to include both women who had delivered in facilities and in their homes, and to represent a range with regard to geographic distance from a formal health facility.

A total of 36 in-depth interviews were held with recently delivered (i.e. within the prior 12 months) women (N = 24), and trained (N = 6) and untrained (N = 6) traditional birth attendants. Of the recently delivered women, 16 had delivered in a facility and 8 had delivered at home. Five focus groups were conducted: two with groups of recently-delivered women, one with women living near (8 participants) and one with women living far from the hospital (8 participants), two groups of traditional birth attendants (15 participants total), and one group of traditional healers (*ng’angas*) and community elders (8 participants). This sample size was selected to ensure a sufficient number of respondents in each subgroup after stratification and is consistent with sample sizes in similar qualitative inquiries [24, 25].

Interviews were conducted in English or Chitonga by a member of the research team and lasted approximately one hour each. They were held at private locations: either the participant’s house, an empty room in the hospital or a private area of the research building. Focus groups were facilitated by a bilingual member of the research team, with a bilingual note taker, were held in private rooms at the Macha research center and lasted two hours each. Participants were compensated for their travel costs and provided with food and beverages. Questions included information about skin care, infection control and thermal care.

Observations of home-based care practices were conducted with families (N = 8) for two-hour sessions. All observations were done within one week of birth, during daytime hours. The observer, a member of the research team, watched silently, taking notes on behavior and not interfering with normal practice.

All interviews and focus groups were audio-recorded. Data were transcribed and translated by bilingual staff and managed using Atlas.ti software. Data were coded by the first author and two research assistants, using thematic analysis techniques [34]. Broad themes were identified *a priori,* based on the field guides, and additional codes were developed during data analysis. A glossary was created for local terms with no direct translation and idiomatic expressions were confirmed with bilingual senior researchers. Findings were reviewed by the research team regularly and data were shared with key informants for confirmation, clarity and quality assurance. The two research assistants were female, had children, and did not have prior relationships with any of the respondents.

Ethical approval was granted by the Institutional Review Boards of the Johns Hopkins Bloomberg School of Public Health and Macha Research Trust. Each participant participated in an oral consent process. Approval for conducting the interviews was also granted by the local chief in each Zambian district and the headmen in each village.

## Results

### Participants

Respondents (n = 75) ranged in age from approximately 18 to 59 years, although many older women were unable to give an exact age. The majority of women, TBAs and elders in the study had less than a secondary school education, and only two respondents had some post secondary school education, both of whom were from villages closest to the Macha Hospital. Trained TBAs had received approximately six weeks of training from the government, although no trainings had taken place since 2002. Trained TBAs were based out of rural health posts and had some access to basic supplies such as latex gloves in limited and irregular quantities. The majority of respondents identified as a member of the Batonga ethnic group and spoke Chitonga.

### Skin care

Skin care practices were often overlapping and related to thermal care practices. Practices varied between newborns perceived to be “regular” and those perceived to be “small” or “sick.” It was commonly reported that a newborn could be identified as “healthy” or “sick” at birth. A healthy newborn is expected to have smooth skin with high elasticity, a loud cry and have strong muscle tone (actively move their arms and legs). Newborns who are lethargic or inactive are considered “weak” (*kubula nguzu*).

A number of substances were reported to be applied to the newborn’s skin by mothers immediately after delivery. Baby lotion, which is commercially available, was the most common, although a few mothers reported that the cost (~4 USD) was prohibitive. Petroleum jelly was often used and can be purchased at local stores at a slightly lower cost than baby lotion. Some mothers disliked petroleum jelly because they stated that it caused a ‘watery rash’ that can only be treated with an injection at the hospital. Those living near the hospital reported more use of lotion or petroleum jelly, which can be bought from the store on the hospital compound, although even these women noted the high cost was a disincentive from using purchased materials. The least commonly reported item that was bought and used was glycerine lotion, and many women stated that it was not as effective at making the newborn’s skin soft. A small percentage of women reported that when they were unable to buy lotion or petroleum jelly, they used cooking oil,^1^ which was reported to be warmed and applied topically, and can be bought at local markets and is often reused. All of the women in the focus groups who were asked to demonstrate on a doll how items were applied to the skin showed that the emollients are applied to the entire face and body. Observers noted that this was done in a gentle manner, although care was not taken to protect the newborn’s mouth, eyes or nose from the lotion.

“You see, we use lotion when we change them… from the head, the feet we do this. This … can keep their skin beautiful.” (Focus group, Recently-delivered woman)

Breast milk was widely reported to be used to make the newborn’s skin softer and was applied to a newborn’s nostrils, vagina or under the foreskin. It was thought to help the newborn urinate and breathe better because the passageways become smoother. Although less widely reported, breast milk was also used as a salve for burns on newborn’s skin.

Respondents understood the vernix to be made of fat. For newborns considered to be “healthy” and full-term, mothers reported wiping the vernix off relatively quickly, under the assumption that the vernix is not needed by term infants. Women commonly waited, however, until they were able to bathe the infant in warm water, and then applied a substance to the skin, and immediately dressed them and wrapped them in blankets. For infants born to HIV-infected mothers, this fat is considered to be a bodily fluid that is diseased and is washed immediately, with urgency, before it is absorbed into the skin. HIV-exposed newborns are washed as soon as possible after delivery, using either cold or warm water. Many of the participants expressed concern about rapid transmission of the virus through the newborn skin.

“You should wash the baby fast. The baby cannot get dirty or keep the sick fluid.” (Interview, Traditional Birth Attendant)

“The fat [vernix] seeps into the skin. That is why you have to wash them so fast. The fat has the virus in it. It is dangerous.” (Interview, Traditional Birth Attendant)

Newborns are locally identified as “preterm” (*mayaba*) if born before the mother has missed nine menstrual cycles. All respondents agreed that it is beneficial for small newborns to have extra fat, with the understanding that the fat from the vernix can seep into the skin if not washed. Therefore, preterm newborns are wrapped immediately and not bathed unless the mother is known to be HIV-infected. Retaining the vernix and covering the skin is thought to facilitate absorption of the vernix into the skin during the first week of life.

“If you bath[e] them, they will lose the fat. If you bath[e] them too early, they will lose their skin. The fat is good for them to get into the skin. They are just too fragile when they are preterm.” (Interview, Traditional Birth Attendant)

**Fig. 1:**
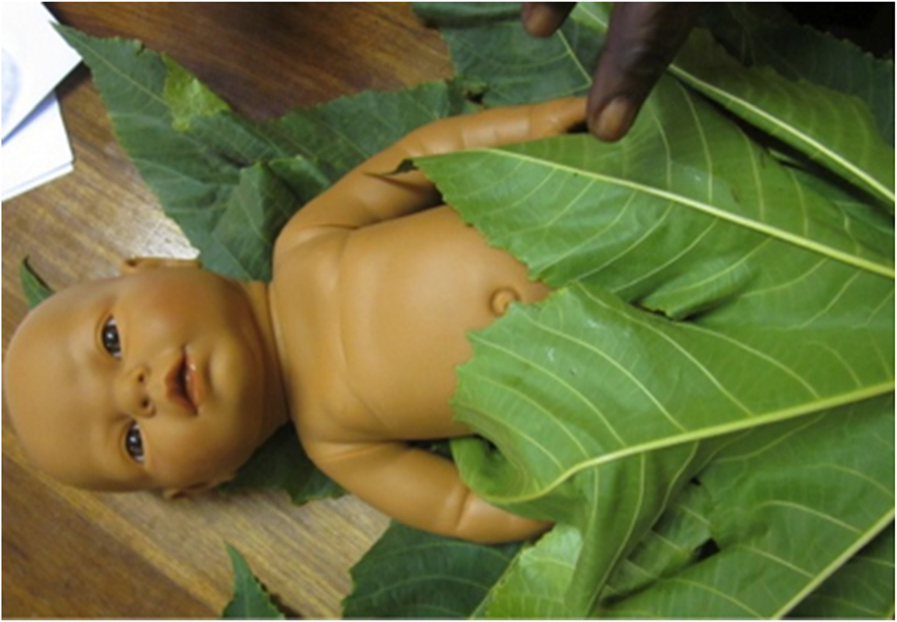
TBAs demonstrating how to wrap a preterm newborn in *mabono* leaves for warmth, using a plastic training doll

To protect the skin, all of the recently-delivered women of preterm infants reported putting *mabono* (castor oil plant) leaves on the newborn’s skin for warmth for at least a month or until it “looks full-term.” These leaves are large enough to cover the torso of the newborn (Fig. 1). For preterm infants, who are not bathed immediately, the vernix keep the leaves stuck to the newborn. Women reported that the same *mabono* leaves can be used for up to two weeks without having to replace them. A few of the mothers also reported putting oil on the skin under the leaves.

“The oil makes the baby warm, so I put the oil many times per day. I rub all over the body so all the skin is covered and his skin is shiny, but I always cover him again with *mabono* so he will stay warm and get bigger.” (Interview, Recently-Delivered Woman)

Additionally, for newborns perceived to be small, leaves from the *mabono* plant may be mashed into the petroleum jelly and the emollient put directly on the skin, all over the newborn’s body. These leaves are thought to contain an ingredient to help make the newborn “fat.” Less commonly, but considered to have the equivalent effect, the same type of leaves were reported to be put into water for the infant to drink, put in a pouch to hang around the newborn’s neck or waist, or crushed and pressed into a small incision in the newborn’s skin. This process often creates permanent keloid scarring and is locally referred to as “tattooing”. Application of emollient to the skin was observed, but tattooing was not, although one newborn had scars on her arms where this had been done in previous days.

A more traditional, but less common, method for treating a preterm infant’s skin is to make an “oil” out of *mabono* pods by frying the pods, extracting the white tissue or beans, pounding it into powder, adding it to water, and then boiling the mixture. A few of the women responded that their mothers or female relatives had made this for them ahead of the delivery since, unlike certain oils, it can be kept in a bottle and applied to the skin later without warming or preparation. Younger women were less aware of how to prepare the oil, but were equally willing to use it.

### Thermal care

Newborns, especially their heads, were kept covered. The neonatal fontanel (*caamutwe*) is widely considered to be an important channel of communication between the newborn and ancestral spirits, and every participant stated that no matter the environmental temperature, all newborns should wear hats all the time to protect the fontanel both from injury and from the malevolent intents of other individuals. This was a common statement across all sites and regardless of the mothers’ level of education or proximity to a facility. Thus, newborns wear hats inside and outside (except while being bathed) for warmth but also to protect the fontanel.

“Babies breathe through that spot [fontanel], for about one and six [one year and six months]. It is very important. If the spot closes too soon, the baby will suffocate and die and no one can help it.” (Interview, Traditional Healer)

Reports and observations confirmed that newborns and infants are generally clothed most of the time. The most commonly observed activities were feeding practices, although some bathing and swaddling practices were also noted. In addition to hats, newborns are clothed with socks on both feet and hands and wrapped in blankets. This was observed for all newborns. Women reported that if they only had one set of newborn clothing, they would dress the newborn in the oversized clothing of an elder child while washing the newborn set. Women without dedicated newborn clothing reported using child or adult shirts and then wrapping the child with traditional cloths or blankets as well. For newborns considered “weak” (*kubula nguzu*), respondents described using warmed, dry cloths as a compress on the newborns joints every day. This is thought to provide strength and restore fluid movement to the newborn’s joints.

Newborns are usually bathed twice a day, in the morning and before sundown, although some variation in the frequency of bathing was reported, with less frequent bathing when the mother had strenuous work or long work hours, such as harvesting. About half of the respondents expressed difficulty in bathing their infants as often as they would have liked. Women who lived further from water sources expressed more concerns about their ability to get water to use for bathing and reported bathing the newborn less often. Women stressed the importance of using warm or tepid water for bathing the infant, except for the ritual first bath after the umbilical cord separates, when the newborn is washed with cold water, as this is thought to be the moment when they are no longer fragile and thus strong enough to withstand a small challenge. Additionally, some mothers and TBAs reported a practice of washing the newborn at night with cold water.

“If the baby wakes up and shivers during the night, he should be washed in cold water, to make the shivering stop. This is good training and it will make him strong.” (Focus Group, Traditional Birth Attendant)

This nighttime bathing was only practiced for full term infants and was more common with male newborns, for whom strength during adversity is seen as a virtue. Newborns are usually bathed immediately after birth, but there is some evidence of changing trends since clinics are encouraging delayed bathing. Some mothers reported waiting a few hours for the first bath, although all stated that newborns should be bathed before nighttime so the newborn is clean while sleeping. Women reported wanting the newborn to be comfortable overnight, which meant keeping them clean and dry and also placing them prone on a soft mat. Observers noted that bathing before sundown generally took place in a warm part of the home, but if no bed were available, newborns may be placed on a mat on the floor during the process.

Participants who lived closer to the hospital reported hearing more messages about delayed bathing and reported waiting a few hours longer to bathe the newborn, but none of the mothers were willing to wait until the following day for the first bath for a full-term infant. All respondents stated strongly that they would not delay bathing for an HIV-infected infant even by hours because retaining the vernix is thought to increase the risk of transmission to the newborn.

### Umbilical cord care

The umbilical cord (*kakombakombo*) and the placenta (*camacembele*) are considered important after the delivery because of their spiritual connection to the newborn. Many respondents discussed the importance of disposing of or burying both the stump of the umbilical cord and the placenta in safe places where others could not find them. The cord and placenta are considered an extension of the newborn and individuals wishing harm upon the newborn could use the parts in rituals to “curse” the child.

Every respondent stated the importance of clean cord care after delivery. Many TBAs stated that in the past any razor blade was used but because of education about clean deliveries they now try to buy a new one. However, a few reported that, especially if it was an early delivery with no time to prepare, they still used an old blade despite the risks. The use of older blades was more common in the more isolated villages, which were further from accessible markets.

“You do not know when the birth is coming. You don’t control it, it just chooses. It just happens and you don’t know when. You want to be ready, but sometimes there is no time.” (Interview, Traditional Birth Attendant)

If the mother’s HIV infection status is known, TBAs stated that they will not cut the cord and will instruct other family members not to have any contact with the blood, so mothers have to cut the cord themselves. This often results in delayed cord cutting for HIV-exposed newborns.^2^

The appearance of blood clots in the umbilical stump (*bulongo-longo*) is considered an abnormality, and potentially an early symptom of future illness. Newborns with this condition are often taken to a healer, who treats the stump with herbs, or to the hospital.

To accelerate cord separation, mothers reported putting breast milk onto the stump. This is used to make it “rot” and fall off faster.^3^ Participants used both the Chitonga words for “rot” (*kubola*) and “dry out” (*kuyuma*) to describe this process. Both mothers and TBAs stated that the breast should not touch the stump, nose or genitalia of the newborn, but rather the milk should be expressed and dripped onto the body part.

Until the cord separates, the mother is expected to apply a wrap around the newborn’s waist. If the cord falls onto the genitals, it is thought that the newborn will be infertile as an adult. Also, mothers reported that the cord is not supposed to fall on the floor and all of the respondents said they paid careful attention to the stump during the newborn’s first days of life.

One of the traditional practices described was more commonly practiced among women living in more isolated areas, further from the hospital, but was recognized by all of the respondents.

“When the stump falls, or in the morning if it was at night, the mother and baby should bath[e] in cold water to make them strong. The small child is called, maybe the daughter or son to the eldest brother. The girlchild will strap [carry] the girl baby or a boychild will strap a boy baby. The boychild will go with an axe to cut a small tree and the girlchild will go fetch water to bring for the family. This is how we show the new baby to be a good man or a good wife.” (Focus Group, Traditional Healer)

This symbolic event, in which the gender roles are modeled and passed to the next generation, is also symbolic as the first time in which the newborn is strapped to one’s back and carried outside of the home. A newborn that is born in the hospital will be carried home sling-style, in front, and is not introduced to the community until after the ritual takes place.

Mothers and TBAs reported wanting the cord to separate quickly, as the newborn is seen as more fragile prior to cord separation. TBAs felt confident to stop visiting once the cord separates and families see this as the time when they can introduce the newborn to the community. Mothers also expressed anxiety that delayed cord separation is symptomatic of a larger problem and will take protective actions to speed cord separation.

To help the umbilicus heal, mothers reported that herbs are rubbed into the stump. For full-term healthy newborns, “black powder” is used, which is made from the burnt stem of a pumpkin. For preterm newborns, mothers reported using “green powder” from the *mweeye* plant. Green powder is made from dried pounded roots that are considered gentler for weak, preterm newborns. Both mothers and TBAs demonstrated how it is put directly on the stump. If the particular herbs cannot be found, brick ash was reportedly used as a substitute. In a tradition that was explained to have originated in Zimbabwe, some mothers reported that fresh dried chicken dung is mashed and put on the wound after the stump falls off. For a female newborn, rooster dung is used; for a male newborn, dried hen dung is used.

## Discussion

In this region of southern Zambia, thermal care practices for newborns are revealed to be largely beneficial, with some significant exceptions. Newborns were generally kept warm with hats and layers of clothing, and extra thermal protection is provided for preterm and small newborns. However, bathing a newborn with cold water during the night could be detrimental. The vernix was considered important for the preterm newborn but dangerous for HIV-exposed infants. Applying harmful substances to the skin and umbilical cord, a commonly reported practice, may amplify exposure to invasive pathogens. Mothers applied various substances to the skin and umbilical cord, most commonly powders made of burnt roots or ash, with special practices for preterm infants. These current practices could facilitate the introduction of umbilical cord cleansing with 4.0 % chlorhexidine application for infants born at home; however, the fact that chlorhexidine delays cord separation may reduce acceptability, given the desire for rapid cord separation.

Women in this study reported a wide range of skin and umbilical cord care practices for home-based newborn care (Table 1), across the spectrum of protective to harmful [17]. Overall, many individuals reported practices indicative of a recognition of the importance of keeping the newborn’s body and head warm, and many used lotion and infant massage, which has the potential to be beneficial for thermoregulation [35], although there is a potential risk of hyperthermia in this environment which warrants further study. The recognition of the importance of warmth could facilitate the introduction of skin-to-skin contact, or “kangaroo mother care (KMC),” in which the newborn is placed on the mother’s bare chest inside her clothing or wrapped fabric. KMC has been shown to be highly beneficial for thermal care, as well as for breastfeeding and respiration [36].

**Table 1:** Local practices and potential benefits and harms to the newborn

Practice	Potential benefit	Potential harm
Wrapping newborns in heavy clothing and applying a hat	Thermal care	Unknown (rashes; hyperthermia)
Immediate bathing	Reduced risk of blood contamination if mother is HIV-infected	Reduced protection from vernix; higher risk of hypothermia
Delayed bathing for preterm infants	Benefit of vernix; reduced risk of hypothermia	
Nighttime bathing in cold water		Increased risk of hypothermia, pneumonia
Substances applied to skin	Potential benefit of newborn massage with baby lotion, petroleum jelly	Unknown effect of use of “cooking” oil (higher potential risk with recycled oil); skin infection e.g. bullous impetigo (possibly from application of contaminated substances such as petroleum jelly from a communal jar)
Application of breast milk to umbilical stump	Unknown effect of breast milk application to stump	Potential risk of HIV transmission if mother is HIV-infected
Application of powders to umbilical stump		High risk of neonatal infection
Protection of fontanel	Protects newborn skull; caregiver sensitivity to sunken fontanel (symptom of dehydration)	
Heated cloth for weak joints	Potential benefit for thermal care and massage	
Variety of cord cutting practices	Use of new razor blades reduces risk of infection	Increased risk of infection from old razor blades
Delayed cord cutting if mother is HIV infected	Potential benefit of additional iron to newborn	
Avoidance of cord blood if mother is HIV infected	Delayed cord clamping; reduced risk of HIV transmission to newborn	
Prone sleep position		Increased risk of sudden infant death syndrome (SIDS)
Retained vernix for preterm or low birth weight infants	Benefit of thermal care and antibacterial benefit	
Wrapping preterm/low birth weight infants in *mabono* leaves	Potential benefit of thermal care	Unknown effect of leaves on skin, especially if old or reused leaves

For all newborns, the World Health Organization (WHO) recommends delayed bathing, immediate covering and drying of the newborn, and maintaining the newborn in a warm area [17]. However, immediate bathing of the newborn is a common practice in many low income countries, with reasons being the need to “cleanse” the newborn of “dirty” or “wet” skin [25, 37]. Bathing may also serve to stimulate the newborn, inducing deeper breaths or stronger cries [37]. Delaying bathing is protective for the newborn, since the vernix has important antibacterial properties [38, 39], and contains the same innate immune proteins as found in breast milk [40]. Delayed bathing of newborns may also help improve thermoregulation. A randomized controlled trial of non-asphyxiated vaginally-born newborns in a referral hospital in Uganda found a reduced incidence of hypothermia among newborns not bathed at one hour after birth [37]. In this study, newborns considered “weak” were reportedly given a treatment to loosen their joints. The use of a dry cloth warmed over a fire on the newborn’s joints also adds to improved thermal protection [17], although more research could be done to understand if there is a specific effect on the newborn’s muscle movements, and if there is any negative effect if the newborn is undressed for a long time during this practice. Additionally, the reported sensitivity to the fontanel can be protective for newborns; awareness of the need to guard and observe the fontanel can aid in thermoregulation and recognition of illness such as dehydration, which can present clinically as a sunken fontanel [41]. There were some significant exceptions to the beneficial practices of thermal care in Zambia. One is the common ritual first bath in cold water after the umbilical cord separates and the other the practice of bathing the newborn in cold water at night, both of which increase the risk of hypothermia and could be harmful.

One of the less common, but potentially most detrimental, practices is that of reportedly placing powders directly into an incision that has been made in the newborn’s skin. The likelihood of bacterial contamination is high and these infections can cause septicemia [17, 42]. Many respondents reported that this practice was, although less common, similar to the practice of placing herbs in a bag and tying it to the newborn’s neck, wrist or ankle. This latter practice likely has minimal, if any, effect on the physiologic health of the newborn and thus would be preferable to incisions.

Of similar concern are the practices of using an unclean blade when cutting the umbilical cord and putting powder or dung on the umbilical stump, both of which can increase exposure to harmful pathogens. Regarding the former, most TBAs were aware that they should use a new razor blade to cut the cord, but reported that consistent supply was a problem. Thus, while the use of unclean or contaminated blades continues to be a threat, the solution lies not in health education as much as provision of appropriate, hygienic materials, which are currently available intermittently and in limited quantities, to trained TBAs. Pregnant women could be encouraged to purchase or provide their own clean razor blade prior to delivery. Putting green or black powder on the umbilical stump was reported to be very common and widespread among all of the respondents but the use of dung was far less common.^4^ In both cases, putting foreign materials on the stump was not considered dangerous and was considered important for accelerating stump healing. Thus, behavior change communication would be an essential strategy to accompany the introduction of new and easily available hygienic materials. Research has shown that effective strategies for behavior change are through mothers’ groups or other peer support groups, where groups that meet regularly can share experiences and learn how to improve on practices [43]. Other possible delivery mechanisms are through health education via community health workers, traditional birth attendants, health worker outreach and mass media campaigns.

The common tendency to apply something to the cord stump might ease the introduction of chlorhexidine cleansing of the cord, which has the potential to reduce neonatal mortality by as much as 23 %, and is now recommended by the WHO in settings with a large percentage of home births [44–46] and is currently being evaluated in trials in Zambia and Tanzania [47, 48]. However, the desire for rapid cord separation may prevent uptake of chlorhexidine, which delays cord separation [28].

Many previous studies have found high awareness among mothers about hypothermia, especially for small or low-birth weight infants; this was found in Tanzania [30], where special attention was also paid to protecting the fontanel, and Nigeria, where palm oil and breastmilk were also used on the skin [49], among other study locations [29, 50]. However, among the Yoruba in Nigeria, more interventions related to herbs and food supplements were provided to small infants [49], whereas in this study in Zambia, there were often fewer interventions for small infants to allow them time to grow. Additionally, in Nigeria, there were similar methods of providing spiritual protection to newborns, including wearing of amulets and making scarifications [49].

Studies in other locations have also noted an emphasis on immediate bathing and, although the specific reasons differed, largely related to the concept of the vernix being “dirty”. In Tanzania, vernix was thought to be sperm and indicated the mother’s sexual promiscuity during pregnancy; the desire to clean the newborn immediately led to resistance to delay of a first bath [30]. In Uganda, mothers expressed concern that the vernix was “smelly” and a “possible source of infection” of HIV [50, 51]. A quantitative study in Ghana found that immediate bathing was common and over three-quarters of newborns were bathed within six hours of birth [29, 52]. In Tanzania, there was also an understanding that bathing the newborn in cold water would make them cry, although bathing at night was not reported [30].

A study in northern Zambia found similar high awareness of hypothermia risk and protective actions with wrapping, drying and multiple layers of clothing [31]. Bathing of preterm infants immediately was not practiced, although the northern Zambia study found a willingness to delay bathing until the day after birth for term infants [31]. That study also described the use of warm water bottles for thermal protection of small or preterm infants, which was not observed in southern Zambia [31].

In general, many of the cord care practices, including cord cutting, in Zambia were reported to be similar to findings in other resource-poor settings, although there was variation in the substances applied [23–25]. The use of powders on the umbilicus was found in to be prevalent in Nigeria [49] but was a less commonly used substance than “hospital medicine” or Shea butter in Ghana [52], and not used in Uganda [50]. In a different study in the southern province of Zambia, actions were taken to accelerate cord detachment and substances such as powder, Vaseline and breastmilk were commonly used, with cooking oil and dung less common. That study also found that, although the benefit of a new razor blade was known, it was not always used [28]. In Ghana, most women reported using a new blade [52]. Also similarly, the Zambian study found concern over clots in the umbilical cord (“*bulongo-longo*”), anxiety over a delayed cord separation and care seeking with traditional healers prior to formal facilities [28]. This study also confirmed previous findings around clandestine locations for burying the placenta and cord stump to protect them from being used for spiritual harm against the infant [28].

Important differences between this study and previous studies relate to care of preterm infants and care of HIV-exposed newborns. Unlike reported practices for preterm newborns in Tanzania, such as immersion of the newborn in cold water [24], the practices reported in this study suggest potentially beneficial home care practices for low birth weight or preterm infants. Although this study did not focus on practices related to infant sleep, it was found that newborns are generally placed in a prone position. This echoes findings from a South African study in the Cape Peninsula where over 60 % of infants were placed in a prone sleeping position [53]. Prone sleeping is known to be associated with a higher risk of sudden infant death syndrome in industrialized countries and many professional pediatric associations now recommend supine sleeping position [54].

Practices for HIV-exposed newborns varied from care for other newborns. Wiping the vernix immediately puts these newborns at higher risk of hypothermia and infection. However, extra care with maternal blood during the immediate postpartum period reduces the risk of transmission of HIV to the newborn. Reluctance of TBAs to cut the cord of an HIV-infected mother meant delayed cord cutting for HIV-exposed infants, which can have potential benefit with regards to neonatal iron status [55]. Given the local understanding that vernix is good for premature newborns, mothers already delay bathing for these infants. However, the understanding of the vernix as containing contaminated fluid is strong and it will likely be difficult to convince HIV-infected mothers to delay bathing of their newborns.

It has been estimated that universal coverage of key behavioral interventions for newborn health could avert a large percentage of neonatal deaths, including up to a 20 % reduction in deaths related to preterm birth complications from just three interventions: delayed bathing, head covering and skin-to-skin care [9]. Behavior change programs should focus both on trying to reduce the most harmful practices and prioritize influencing those behaviors which might be the most amenable to change. In rural Zambia, practices that are potentially the most harmful to the newborn are those that increase the risk of infection, including putting foreign material on the umbilical stump, using unclean blades and making deliberate incisions in the skin for traditional medicinal purposes. The practices which might be the most amenable to change are the dermal care practices, since many individuals already use a wide range of items on the newborn’s skin and may be willing to use other substances, so long as it makes skin soft. Specific care for preterm or low birth weight infants is possible, since the recognition already exists that preterm and small newborns require differential care, including increased thermal protection. More investigation is needed into current home practices for preterm infants, such as thermal care with *mabono* leaves, to determine if these practices are harmful, beneficial or have no impact on the health of these low birth weight or preterm newborns.

### Strengths and limitations

This study was designed using qualitative methods to understand local perceptions and the patterns and organizing logic of behaviors surrounding newborn health practices. Methods were chosen which allowed for in-depth investigation into the research questions. An important strength was the triangulation of methods, employing multiple data collection tools to ensure consistency, quality and accuracy. Unlike some other qualitative studies, this study included observation sessions, which allowed for confirmation of reported practices. The inclusion of participants with various roles in pregnancy and childbirth allowed for corroboration between accounts. This study adds to the limited literature in Africa related to newborn care in the home, especially in relation to HIV, and local perceptions of newborn illness, and has important implications for programs and policymakers.

There were limitations inherent in the study design. Much of the data were descriptive and relied on reported information. These data are subject to recall bias and desirability bias, although attempts were made to mitigate the latter effect through rapport-building, an emphasis on learning rather than judging, and use of multiple sources of data, including observation. As observations were not conducted directly at birth but rather within the first week, conclusions regarding certain behaviors practiced in the first hours after birth (such as wrapping and drying the infant) depend solely on interviewee reports. This study has limited generalizability and is only a preliminary investigation around the prevalence of reported practices. Further quantitative research would be needed to understand the frequency of various newborn care practices.

## Conclusions

Given that community-based research on neonatal care in sub Saharan Africa has emerged relatively recently (relative to South Asia), this study fills an important research gap, addressing newborn care issues where rates of neonatal mortality and morbidity are high and formal obstetric and neonatal care is limited. The findings from this study can help determine the training priorities for caregivers to identify vulnerable and ill newborns, as well as provide insight into how a package of newborn interventions can be best adapted and introduced into this and other similar rural sub-Saharan African communities. The design of effective and acceptable interventions in this setting requires a focus on behavioral change communications that can improve these practices.

## Footnotes

1 The most commonly available type of cooking oil in this region is a mixture, similar to vegetable oil, often made with sunflower seed. It is common for oil to be reused and “recycled oil” is available at local markets.

2 For male newborns, the practice is to cut the cord at the same length as the newborn’s arms (elbow to wrist); this is thought to correspond with future length of the penis.

3 Breast milk is also reportedly applied to the newborn’s nostrils to smooth the skin and make it easier for the newborn to breathe, and on penis, under the foreskin, or in the vagina, to ease urination and to make the newborn “sweet to the opposite sex” as an adult.

4 Prior to the widespread uptake of the antenatal tetanus toxoid vaccine, this practice likely caused many cases of neonatal tetanus.
